# Moving forwards with the Standard THC Unit: what are the next steps?

**DOI:** 10.1192/j.eurpsy.2024.122

**Published:** 2024-08-27

**Authors:** V. Lorenzetti, T. Freeman

**Affiliations:** ^1^Neuroscience of Addiction and Mental Health Program, Healthy Brain and Mind Research Centre, Australian Catcholic University, Melbourne, Australia; ^2^Psychology, University of Bath, Bath, United Kingdom

## Abstract

**Abstract:**

Current metrics of cannabis use are inconsistent. This issue prevents the integration of the literature to date and to robustly measure the health risks and benefits associated with specific levels of cannabis consumption. This talk will overview a number of international initiatives to improve the current metrics of cannabis use.

The Standard THC Unit was created to objectively measure cannabis potency across all products, mode of administration, jurisdictions, contexts and over time.

To build upon the notion of the Standard THC Unit, addional multidisciplinary, international consensus based frameworks have been created.

One such ongoing initiatives, seeks to reach expert consensus on how cannabis potency should be reported in cannabis products in order to clearly and effectively inform consumers. The talk will overview preliminary results of the Delphi.

A similar Delphi methodology was used to establish internationally agreed-upon minimum standards to measure cannabis consumption in research (iCannToolkit), the results of which will be outlined.

Overall, it is imperative for cannabis researchers to join forces with multidisciplinary experts in order to improve metrics of use to inform consumers, general practitioners, researchers and public health experts on the harms and benefits associated with cannabis use.

**Disclosure of Interest:**

None Declared

